# Irrupting prey populations in the absence of a mammalian apex predator drive shifts in prey selection by eagles

**DOI:** 10.1007/s00114-022-01804-x

**Published:** 2022-06-08

**Authors:** Matthew Brun, Amoi Stubbs Oliver, Joel Alves, Alex Nankivell, Mike Letnic

**Affiliations:** 1 Evolution and Ecology Research Centre, University of New South Wales, Sydney, 2052 Australia; 2 CIBIO, Centro de Investigação Em Biodiversidade E Recursos Genéticos, InBIO Laboratório Associado, Universidade Do Porto, 4485-661 Vairão, Porto, Portugal; 3 Palaeogenomics & Bio-Archaeology Research Network Research Laboratory for Archaeology and History of Art, University of Oxford, Dyson Perrins Building, South Parks Road, Oxford, OX1 3QY UK; 4 Nature Foundation, PO Box 34, Prospect, SA 5082 Australia

**Keywords:** Removal of apex predators, Perturbation, Dingoes

## Abstract

Removal of apex predators can have far-reaching effects on the organization and structure of ecosystems. This occurs because apex predators can exert strong suppressive effects on their prey and competitors and perturbation of these interactions can shift the balance of interactions between dyads of species at lower trophic levels and trigger trophic cascades. Dingoes (*Canis dingo*) are Australia’s largest mammalian carnivore. Because they are a pest to livestock producers, dingo populations are suppressed in many regions. Suppression of dingo populations has been linked to a suite of ecosystem changes due to ensuing population irruptions of their prey and competitors. Here, we investigate the impact that the suppression of dingoes has on the diet of wedge-tailed eagles (*Aquila audax*) in Australia’s Strzelecki Desert. Wedge-tailed eagles are generalist predators that readily shift their diet in relation to prey availability. We assessed the abundance of species frequently preyed on by eagles and quantified prey remains at eagle nests located on either side of a dingo-proof fence where dingoes were common and rare, respectively. Wedge-tailed eagles consumed more species where dingoes were rare compared to where dingoes were common. Kangaroos (Macropodidae) and western bearded dragons (*Pogona vitticeps*) were more abundant and were consumed more frequently by eagles where dingoes were rare. Introduced European rabbits (*Oryctolagus cuniculus*) were the prey item most frequently identified at eagle nests. However, rabbits were more abundant and their remains were found at a higher proportion of nests where dingoes were common. Our results provide evidence that shifts in the composition of vertebrate assemblages associated with the presence/absence of dingoes, particularly the irruption of kangaroos, influence the diet of wedge-tailed eagles. More generally, by showing that the presence/absence of dingoes can influence the diet of wedge-tailed eagles, our study highlights how pervasive apex predators’ effects on ecosystems can be.

## Introduction

Removal of apex predators can shift ecosystems to alternative states. This shift occurs because apex predators can exert strong suppressive effects on their prey and competitors and perturbation of these interactions can in turn trigger trophic cascades by shifting the balance of interspecific interactions between species at lower trophic levels (Estes et al. 2011). Where apex predators are removed from ecosystems, their herbivore prey and smaller predators (mesopredators) typically irrupt due to relaxation of their top-down effects (Prugh et al. 2009). In turn, increased impacts of irrupting herbivores and mesopredators may have cascading impacts on species at lower trophic levels and result in the depletion of plant biomass and suppressed populations of small prey species, respectively (Ripple et al. 2014).

Because they exert their top-down effects via multiple interaction pathways, removal of apex predators can affect a vast number of species and ecological processes, often in unexpected ways (Estes et al. 2011; Ripple and Beschta 2004). For example, suppression of small prey in areas where mesopredators have irrupted can suppress populations of avian predators by reducing the availability of their prey (Lee et al. 2021; Rees et al. 2019b). Shifts in vegetation structure due to changes in the intensity of herbivory in the absence of apex predators can influence how water and wind flow across landscapes and drive changes in the geomorphology of landscapes (Beschta and Ripple 2006; Lyons et al. 2018).

Australia’s largest predator is the dingo (*Canis dingo*) (Crowther et al. 2014) which replaced the Tasmanian tiger (*Thylacinus cynocephalus*) as the mainland continent’s apex predator between 3000 and 5000 years before present (Letnic et al. 2012). Dingoes are considered to be a pest by many farmers because they kill livestock, particularly sheep. Consequently, intensive effort is spent on suppressing dingo populations in many regions (Letnic and Crowther 2020). The most widely used methods to suppress dingo populations are exclusion fencing, poisoning, trapping and shooting (Fleming et al. 2001). Exclusion fences have been particularly effective at reducing dingo populations and work by preventing immigration of dingoes into areas where their populations have been suppressed (Fleming et al. 2001). The longest of these fences, the dingo barrier fence, is > 5000 km in length (McKnight 1969). This fence traverses the semi-arid and arid rangelands of southern Australia and has a distinct effect on human economic activity in the rangelands (McKnight 1970).

Dingoes are uncommon on the southern and eastern sides of this fence, enabling the widespread grazing of sheep (McKnight 1970). Dingoes are relatively common and only subject to sporadic control on the northern and western side of the fence, termed the outside of the fence. Cattle are grazed primarily on the outside of the fence (McKnight 1970).

**Fig. 1:**
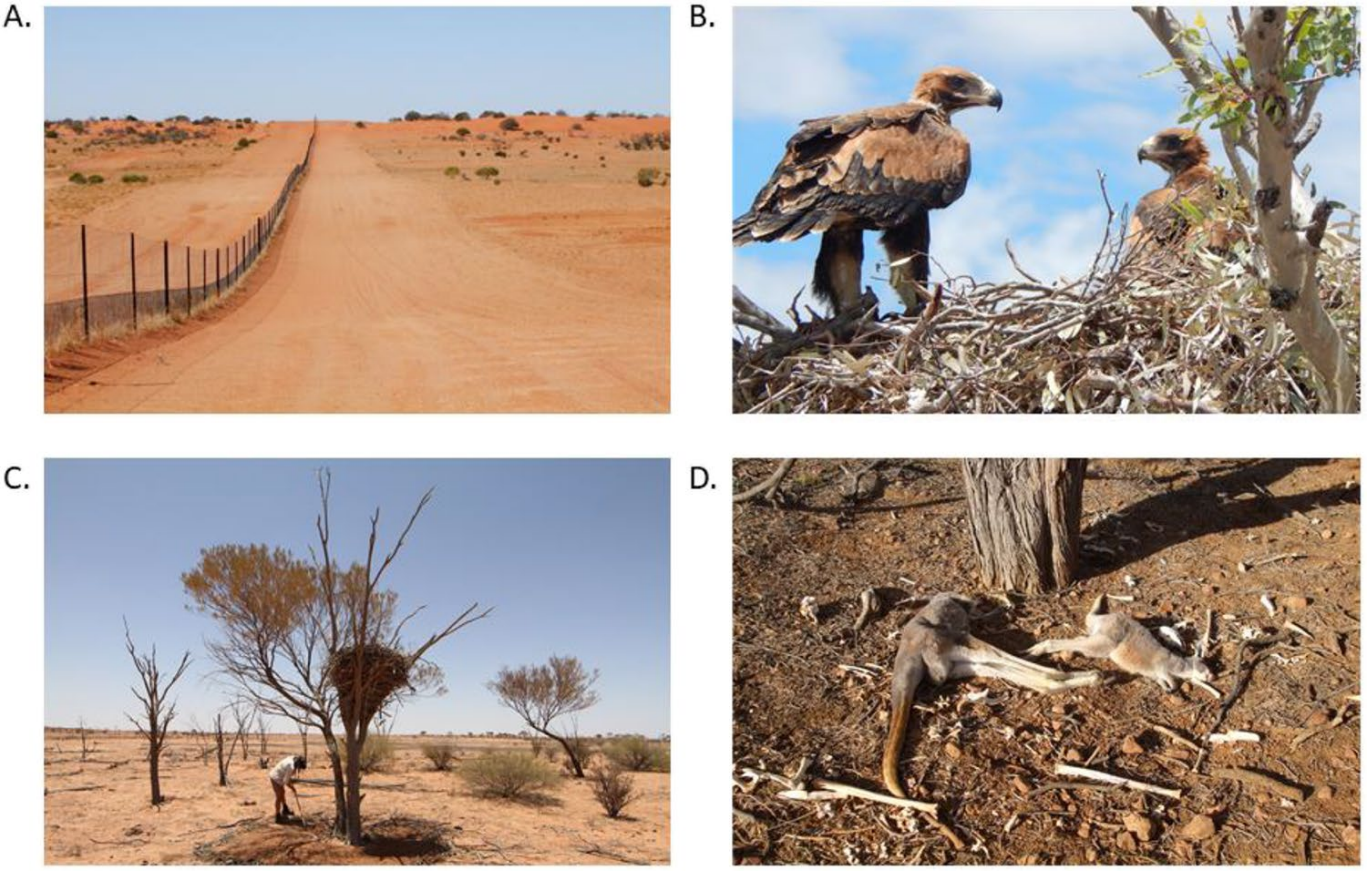
The dingo fence along the border of NSW and SA (**A**), Fledgling wedge-tailed eagles in a nest in NSW (**B**), Nest being surveyed for prey remains in South Australia (**C**) and red kangaroo with young at foot that died in the mass mortality event in 2018 (**D**)

By suppressing dingo populations, the dingo barrier fence also has a profound effect on the ecology of arid Australia (Fig. 1a) (Letnic et al. 2009). Indeed, the impacts of dingo removal in the Strzelecki Desert region were so stark that the ecologist Alan Newsome described the two sides of the fence as being separate ecological universes (Newsome et al. 2001). In this region, the dingo fence demarcates the borders of the states of NSW and SA and NSW and Qld. In areas where dingoes have been excluded/suppressed, populations of kangaroos, emus (*Dromaius novaehollandiae*), introduced red foxes (*Vulpes vulpes*) and feral cats (*Felis catus*) have irrupted (Caughley et al. 1980; Feit et al. 2019; Letnic and Koch 2010). The irruption of mesopredators has in turn been linked to suppressed populations of small mammals, rabbits and varanid lizards, and shifts in the composition of assemblages of birds and arthropods (Contos and Letnic 2019; Feit et al. 2019; Gordon et al. 2017b; Rees et al. 2019a). Similarly, shifts in the composition and architecture of plant communities across the dingo fence have been attributed to increased herbivory by kangaroos, decreased granivory by rodents and decreased herbivory by rabbits in areas where dingoes have been excluded (Fisher et al. 2021; Gordon et al. 2017a).

Wedge-tailed eagles (*Aquila audax*) are the largest bird of prey in Australia and are common in arid regions of the continent. These birds are generalist predators that readily shift their diet in response to the availability of prey (Brooker and Ridpath 1980; Leopold and Wolfe 1970; Olsen et al. 2014; Parker et al. 2007; Silva and Croft 2007). In arid Australia, eagles prey heavily on rabbits and reptiles but also prey on larger species such as kangaroos and sheep (Brooker and Ridpath 1980; Fuentes and Olsen 2015; Sharp et al. 2002b). Wedge-tailed eagles frequently consume carrion (Leopold and Wolfe 1970; Newsome and Spencer 2021; Rees et al. 2020). When adequate food is available, wedge-tailed eagles breed in the austral winter–spring and attend large nests constructed of sticks (Debus 2017; Ridpath and Brooker 1987; Sharp et al. 2001). These nests provide an opportunity to study the diets of eagles because prey remains accumulate under the nests and nearby perches (Ridpath and Brooker 1986; Sharp et al. 2002a).

In this study, we compare assemblages of prey remains at the nests of wedge-tailed eagles and abundance of their prey species on either side of the dingo fence in the Strzelecki Desert. We expected that the diet of eagles would differ across the fence due to differences in the availability of their prey. In particular, we expected that eagles’ consumption of kangaroos would increase in the absence of dingoes because kangaroo populations have irrupted where dingo populations are suppressed (Caughley et al. 1980; Fisher et al. 2021). To investigate whether the abundance of prey for eagles differed on each side of the dingo fence, we calculated indices of the abundance for three key species consumed by eagles: rabbits (*Oryctolagus cuniculus*), red kangaroos (*Osphranter rufus*) and a medium-sized lizard, the western bearded dragon (*Pogona vitticeps*) over a 4-year period preceding and overlapping the period when prey items were collected from nests.

## Study area

**Fig. 2:**
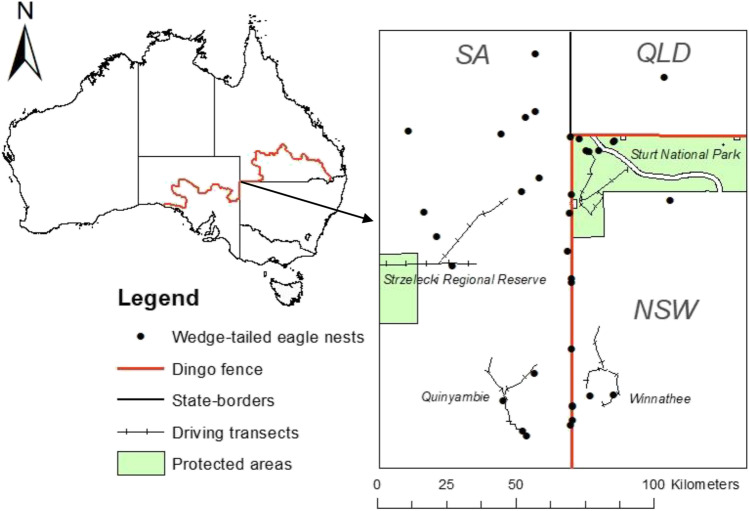
Map of the study region showing the location of eagle nests and driving transects

The dingo barrier fence was erected during the early twentieth century and extends for over 5000 km through New South Wales (NSW), Queensland (QLD) and South Australia (SA)(McKnight 1969) as shown in Fig. 2. We conducted our study on each side of the section of dingo fence that delineates the borders of the states of New South Wales and South Australia and the borders of New South Wales and Queensland, respectively. The NSW/SA border follows the meridian 141° E and the NSW/Qld border follows the parallel 29° S. Thus, these sections of fence take an arbitrary path with respect to the physical landscape. Dingoes are rare on the NSW side of the fence due to intensive efforts undertaken to kill dingoes through trapping, shooting and poisoning and because the existence of the fence prevents immigration from areas where dingo populations are not controlled. In contrast, dingo populations are only controlled sporadically on the South Australian and Queensland sides of the fence. Hereafter, the NSW side of the fence will be termed as inside the fence and the SA and Qld side of the fence will be termed as outside the fence.

The landscape of the Strzelecki Desert is characterized by east–west longitudinal sand dunes interspersed with clay-soil interdunal swale zones. Mean annual rainfall in the study region is less than 250 mm. Vegetation in the study region is characterized by a sparse overstorey of perennial shrubs (*Acacia aneura*, *Acacia ligulata*, *Dodonaea viscosa* subsp. *angustissima*) and a short (< 40 cm) understorey of ephemeral grasses (*Aristida contorta*, *Eragrostis* spp., *Sporobolus actinocladus*) and forbs (*Sclerolaena* spp., *Portulaca oleracea*, *Salsola australis*). The study was conducted on pastoral properties and two conservation reserves, Sturt National Park (29° 9′ S, 141° 20 ʹ E) and Strzelecki Regional Reserve (29° 24ʹ S, 140° 33ʹ E) situated on either side of the dingo fence in the Strzelecki Desert. Assessments of prey abundance were conducted at 2 sites on each side of the dingo fence in NSW and SA, respectively. The sites where dingoes were rare in NSW were Sturt National Park and the pastoral station Winnathee (29° 45ʹ S, 141° 60ʹ E). The sites where dingoes were common in SA were Strzelecki Regional Reserve and the pastoral station Quinyambie (29° 50′ S, 140° 46′ E).

## Prey assemblages at eagle nests

On four sampling occasions between June 2018 and March 2019, we located 36 eagle nests and collected prey remains from directly under the nest and perches within a 30-m radius of the nest. Searches were undertaken by searching the ground and scouring through litter and were conducted for approximately 0.5 person hour. Remains of all prey items located were bagged and taken back to the laboratory where they were identified. For mammals and reptiles, prey remains were identified to species level using a reference collection. We were unable to identify bird remains to species level. Hence for the purposes of tabulation and analyses, these remains were classified as bird. Because we did not know either the time frame over which prey had accumulated at nests or when eagles had last occupied nests for the purposes of tabulation and analyses, we simply noted prey taxa as being present or absent at nests. We then calculated the frequency of occurrence of prey items on each side of the dingo fence as the number of nests at which prey items were located divided by the total number of nests that were searched.

## Abundance of prey species

We surveyed for rabbits by spotlighting whilst driving transects (15–30 km) at night, between 19:00 and 24:00. Surveys were conducted on occasions between March 2015 and March 2019. At each site on each survey occasion, spotlight surveys were always conducted along the same transects (see Fig. 2). During surveys, we drove at a constant speed of 15 km/h, whilst an observer scanned the landscape with a 50 W spotlight from the roof of the vehicle. Because we rarely sight rabbits that are > 50 m from the vehicle, we only recorded rabbits that were estimated to be within 50 m of the vehicle. We conducted 2–3 spotlight surveys (7–20 km long) at each study site on the same roads on each sampling occasion. On each sampling occasion, the density of rabbits was counted along ≥ 30 km of track. The density of rabbits (i.e. number of rabbits sighted km^−1^) at each site on each survey occasion was calculated by dividing the total number of rabbits sighted by the area surveyed and distance surveyed (length of transect multiplied by 0.1 km strip width).

At each study site, we conducted driving surveys in the late afternoon to estimate the density of red kangaroos. Surveys were conducted on 13 occasions between March 2015 and March 2019. On each survey occasion, kangaroos were counted along belt transects by driving 15–20 km/h along single-lane unsealed roads with an observer standing on the back of a 4WD vehicle or observing from either side of the vehicle. Because most kangaroos were observed within 100 m from the vehicle, we restricted our counts to individuals that were < 100 m from the road. On each survey occasion, we conducted 2–3 afternoon surveys (7–20 km long) at each area on the same roads on each sampling occasion ≥ 30 km of track. For each survey occasion, the results from multiple surveys conducted along different tracks were pooled to give a single value. For analyses, the density of kangaroos at each site on each survey occasion was calculated as the number of kangaroos observed divided by the area surveyed (length of transect × 0.2 km width of transect). To provide an indication of the extent of the kangaroo mortality event that occurred in 2018, we recorded dead kangaroos (carcasses and skeletons) that were within 100 m of the vehicle during the kangaroo survey conducted in Sturt National Park in June 2018 and estimated their density using the method described above for live kangaroos.

The abundance of bearded dragons was indexed by pitfall trapping at each site during autumn and spring between 2015 and 2018 and in autumn 2019. At each site, 7 or 8 trapping grids were established. Each grid comprised 9 pitfall traps arranged in three lines of three traps, spaced 50 m apart. Each pitfall trap was constructed from PVC pipe (length: 600 mm, diameter: 150 mm) buried vertically, such that the tops were flush with ground level. Each trap had a 10 m length of drift fence constructed of aluminium flywire (height: 200 mm) positioned over the trap. The bottom of the pit traps was fitted with flywire mesh to prevent captured animals burrowing free. On each sampling occasion, traps were opened for two to four consecutive nights and checked each following morning. We processed captured animals at the site, marking them on the base of the tail with a felt-tip marker pen before releasing them. We determined the relative abundance of bearded dragons by dividing the number of individuals captured at a site on each survey occasion by the number of grid nights performed at each site. The number of grid nights was calculated by calculating the sum of the number of nights that each grid was open for.

## Analyses

A generalized linear model was used to compare the number of prey items found at nests on each side of the dingo fence. Because the data were counts, a Poisson distribution was used.

A generalized estimation equation (Zuur et al. 2009) assumes a Gaussian distribution was used to investigate the effects of dingo fence, sampling period and the interaction between dingo fence and sampling period on abundance indices of kangaroos, rabbits and bearded dragons. Models used a Gaussian distribution because the abundance indices were expressed as rates. Because each site was subjected to repeated measures, sampling trip was included as a repeat using an autoregressive (AR1) term to account for the autocorrelation of the counts through time. The AR1 correlation structure considers that a value of the response variable at sampling period *t* is correlated with a value of the response variable at sampling period *t-1* (Zuur et al. 2009).

We compared presence-absence data for each prey category at each nest using non-metric multi-dimensional scaling (nMDS) plots based on Bray–Curtis similarity matrices for each site. We then used ANOSIM (analysis of similarities) to determine if prey assemblages at eagle nests differed on each side of the dingo fence (Clarke 1993). If a significant result (*P* < 0.05) was obtained using ANOSIM, we used the SIMPER procedure to determine which prey taxa contributed > 10% to differences in the prey assemblages at eagle nests (Clarke 1993). We used PRIMER 5 for Windows (version 5.2.4) for all analyses (Clarke and Gorley 2001).

## Results

### Prey assemblages at eagle nests

**Fig. 3:**
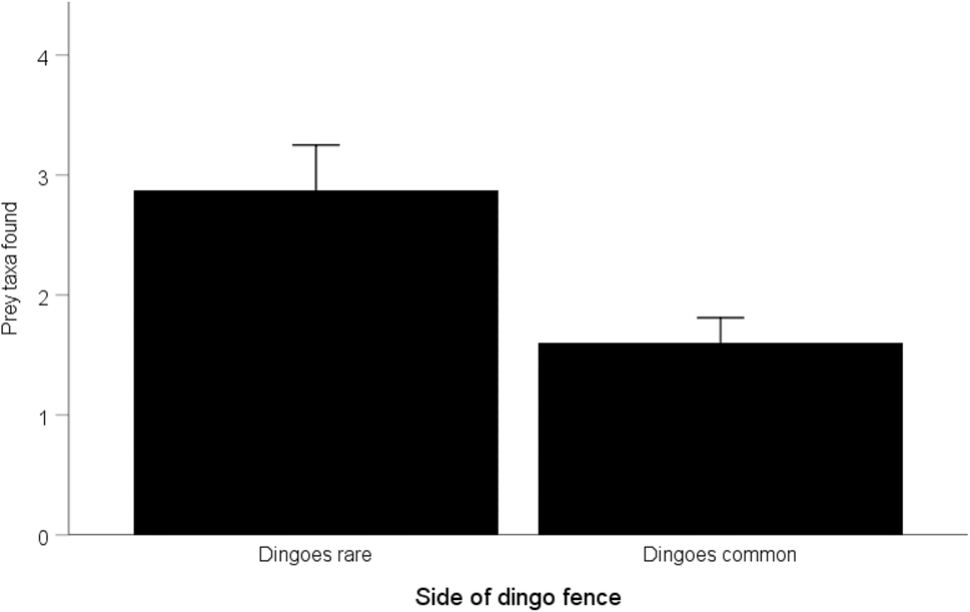
Mean number of prey taxa found at eagle nests where dingoes were common and where dingoes were rare. Error bars represent 1 standard error

Eagle nests on the side of the fence where dingoes were rare had a greater number of prey types than nests where dingoes were common (Fig. 3; Wald 6.482, df 1, *P* = 0.011). Rabbits were the most frequently occurring prey item at eagle nests on both sides of the dingo fence and were by far the most frequently found item at nests on the outside of the dingo fence (Table 1). Kangaroo remains were only found at nests on the inside of the dingo fence and were found at 81% of nests on the inside of the dingo fence. Bearded dragons and shingleback skinks (*Tiliqua rugosa*) were found at nests on both sides of the dingo fence. Other prey items observed at low frequencies were unknown bird species, dingo, fox, feral cat, central netted dragon (*Ctenophorus nuchalis*), sheep (*Ovis aries*), unknown snake species and sand goanna (*Varanus gouldii*).

**Table 1:** Proportion of nests with taxa *n* = 16 inside, *n* = 20 outside

Taxon	Common name	Dingoes rare	Dingoes common
*Oryctolagus cuniculus*	Rabbit	0.81	0.95
*Osphranter rufus*	Red kangaroo	0.81	0
*Felis catus*	Feral cat	0.06	0.05
*Vulpes vulpes*	Red fox	0.06	0
*Canis dingo*	Dingo	0	0.05
*Ovis aries*	Sheep	0.06	0
*Tiliqua rugosa*	Shingleback	0.3	0.1
*Pogona vitticeps*	Bearded dragon	0.56	0.3
*Ctenophorus nuchalis*	Central netted dragon	0.06	0
*Varanus gouldii*	Sand goanna	0.06	0
Snake		0.06	0
Bird		0.06	0.15

**Fig. 4:**
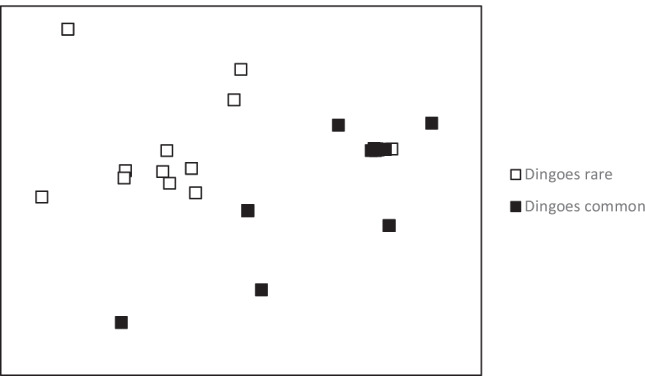
nMDS ordination comparing similarity of prey remains found at eagle nests on each side of the dingo fence. Presence/absence data, Bray–Curtis similarity matrix, stress = 0.07. Less than 36 symbols are presented because points overlap.

Ordination and ANOSIM (*R* = 0.365, *P* < 0.001) analysis showed strong separation in prey assemblages at nests on each side of the dingo fence (Fig. 4). SIMPER analysis revealed that kangaroos were the principal driver of the separation in eagle diet on each side of the fence, followed by bearded dragon and rabbit (Table 2).

**Table 2:** SIMPER presence/absence. Average dissimilarity 55.14. Only species that contributed more than 10% to the dissimilarity solution are presented

Prey species	Average dissimilarity (%)	Contribution to the dissimilarity solution (%)
Kangaroo	18.84	34.16
Bearded dragon	11.58	21.00
Rabbit	7.13	12.94

## Abundance of prey species

**Fig. 5:**
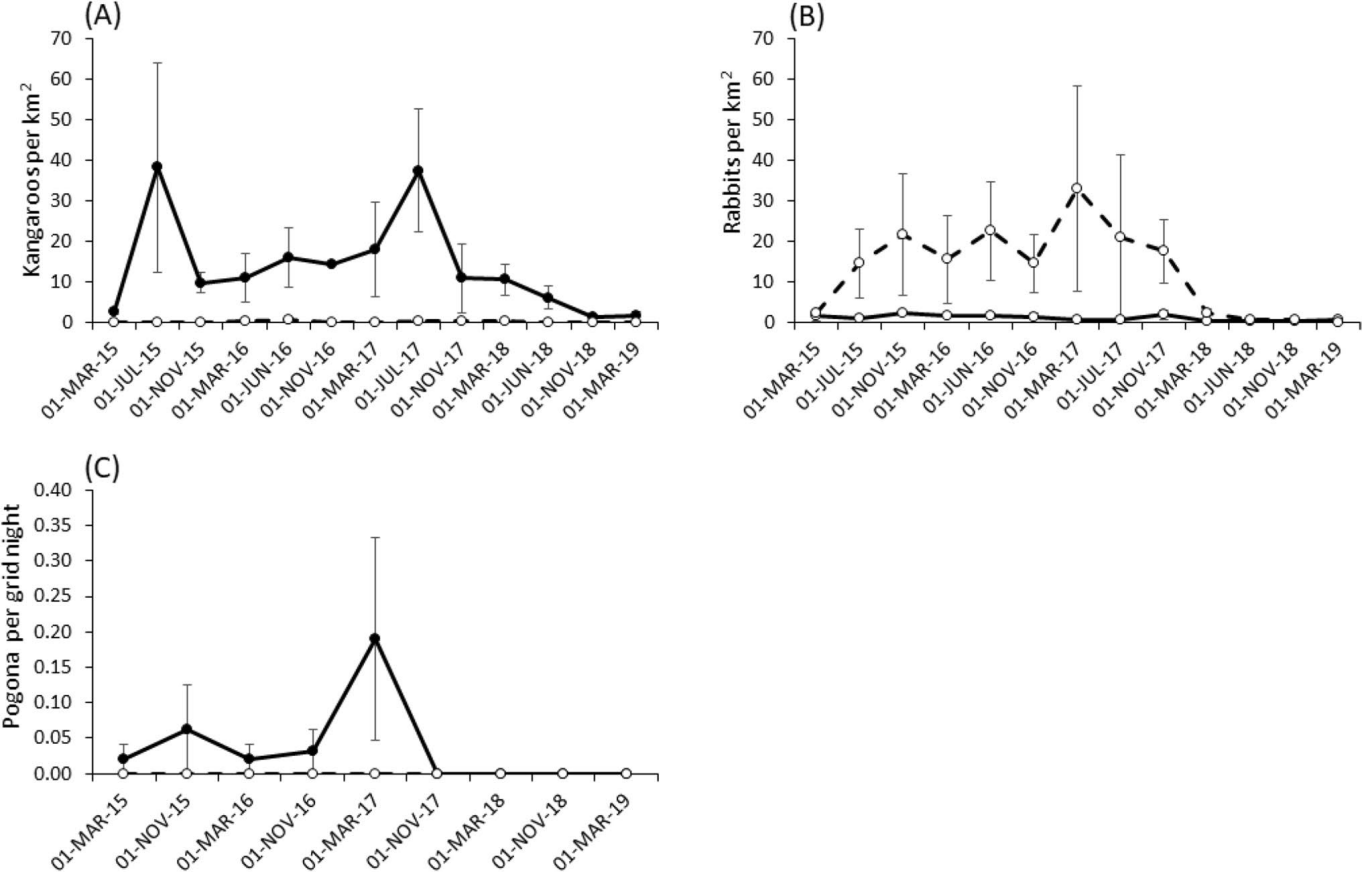
Abundance indices for eagle prey species **A** kangaroos, **B** rabbits and **C** bearded dragons between March 2015 and March 2019

There were effects of fence (Wald *χ*^2^ = 19.633, df 1, *P* < 0.001), time (Wald *χ*^2^ = 14,777, df 2, *P* < 0.001) and the interaction between time and fence (Wald *χ*^2^ = 14,098, df 2, *P* < 0.001) on kangaroo abundance. Kangaroos were more abundant on the inside of the fence and their abundance fluctuated through time (Fig. 5A). Kangaroos were rarely sighted where dingoes were common. Where dingoes were rare, kangaroo density exhibited peaks in July 2015 and July 2017. After July 2017, kangaroo density declined markedly. During the March 2018 and June 2018 sampling trips, we witnessed numerous kangaroos dying, apparently of starvation. The density of dead kangaroos (Fig. 2D) recorded during driving transects conducted in Sturt National Park in July 2018 was 22.3/km^2^. Eagles were observed killing moribund kangaroos and feeding on dead kangaroos during this sampling trip.

Rabbits were more abundant where dingoes were common (Fig. 5B). The density of rabbits varied through time (Wald χ^2^ = 7.584e^10^, df 2, *P* < 0.01) and across the dingo fence (Wald *χ*^2^ = 4.197, df 1, *P* = 0.040), and there was a significant interaction between fence and sampling trip (Wald *χ*^2^ = 8.302, df 2, *P* = 0.015). Rabbit densities fluctuated markedly where dingoes were common but remained at low densities and showed little fluctuation where dingoes were rare (Fig. 5B). Where dingoes were common, the density of rabbits increased markedly after March 2015 and remained at densities above 10 per km^2^ until November 2017. Rabbit densities declined markedly after November 2017.

The abundance of bearded dragons differed across the dingo fence (Wald *χ*^2^ = 9.042, df 1, *P* < 0.003), but was not affected by sampling period (Wald *χ*^2^ = 2, df 1, *P* < 0.157) or the interaction between fence and sampling period (Wald *χ*^2^ = 2, df 1, *P* < 0.157). Bearded dragons were only captured on the inside of the fence (Fig. 5C), although they were observed anecdotally, and their remains located at eagle nests on the outside of the fence (Table 1). Bearded dragon abundance declined after March 2017 and no individuals were captured between November 2017 and March 2019 (Fig. 5C).

## Discussion

Distinct differences in the assemblages of prey remains at eagle nests on each side of the fence corresponded with differences in assemblages of vertebrate fauna during the 4 years preceding collection of prey remains from nests. A greater number of prey types were found at nests where dingoes were rare. Kangaroos were more abundant and their remains were only recorded at nests where dingoes were rare. Bearded dragons were more abundant and were located more frequently at nests where dingoes were rare. Rabbits were the prey item most frequently identified at eagle nests. However, rabbits were more abundant and their remains were found at a higher proportion of nests where dingoes were common. These findings provide evidence that shifts in the composition of vertebrate assemblages associated with the presence/absence of dingoes, particularly the irruption of kangaroos, influence the diet of wedge-tailed eagles.

Whilst our results show distinct differences in prey assemblages at eagle nests and abundance of eagle prey on either side of the dingo fence, our study is not without its shortcomings. We acknowledge that we only collected prey remains from a relatively small number of nests over a short time period and we had no information available on when the nests were last occupied by eagles. Thus, it is possible that our findings on eagle diet may only be relevant to the period just before and during which we sampled eagle nests. A further caveat is that the dingo fence is not a barrier to eagles, and thus, it is possible that prey remains found at eagle nests had been captured on the other side of the dingo fence, particularly for nests located near the fence. Nonetheless, we think it is unlikely that the difference in prey remains at eagle nests across the dingo fence was an artefact of some other factor, because the shifts in the diets of eagles which we report were consistent with shifts in the abundances of eagle prey.

When all nests were considered together, rabbits were the most frequently recorded item at eagle nests. This is consistent with previous studies showing rabbits are the dominant prey items for eagles across much of the continent (Brooker and Ridpath 1980; Olsen et al. 2010; Sharp et al. 2002b). The importance of rabbits in the diet of eagles is emphasized by the fact that along with kangaroo, rabbits were the most frequent prey item at nests where dingoes were rare, even though for much of the study period, rabbits occurred at much lower densities where dingoes were rare. Where dingoes were common, rabbits were by far the most frequently located prey item at eagle nests. However, nests on the side of the fence where dingoes were rare had a greater number of prey types present. This finding suggests that eagles increased their consumption of other prey types where rabbit numbers were lower. The shift in prey assemblages at eagle nest across the dingo fence is consistent with observations showing that eagles’ diets vary markedly across their range in response to the availability of prey (Brooker and Ridpath 1980; Corbett et al. 2014; Harder 2000; Leopold and Wolfe 1970; Olsen et al. 2010; Winkel 2007). Similarly, our finding that rabbits were more abundant where dingoes were common for most of the study period accords with previous studies from this region which have attributed low numbers of rabbits where dingoes are rare to higher rates of predation by red foxes and feral cats (Letnic and Koch 2010; Newsome et al. 2001).

It is well established that kangaroos occur at much higher densities on the inside of the dingo fence in NSW than adjacent areas in South Australia and Queensland where dingoes are common (Caughley et al. 1980; Letnic and Koch 2010; Newsome et al. 2001). Correspondingly, kangaroo remains were frequently found at nests on the side of the fence where dingoes were rare but we found no kangaroo remains at nests on the side of the fence where dingoes were common. We hypothesize that the high frequency of kangaroo remains at nests in NSW was due to eagles both hunting them and scavenging, as we have observed eagles to kill and then consume living kangaroos and to consume kangaroo carrion. Similarly, previous studies have reported eagles to kill kangaroos and to consume kangaroo carrion (Brooker and Ridpath 1980; Fuentes and Olsen 2015; Leopold and Wolfe 1970; Rees et al. 2020).

It is possible that the high frequency of kangaroo remains we observed at nests inside the dingo fence may have been due to eagles feeding on kangaroos that they killed or had died of starvation during the severe drought conditions which prevailed in 2018. During this sampling period, we saw eagles killing moribund kangaroos and feeding on recently dead kangaroos. However, it is important to note that kangaroo mortality due to natural causes and human activities such as vehicle accidents and culling occurs continuously and thus kangaroo carrion, whilst being more available during mass mortality events that coincide with severe drought, may be expected to frequently be available for eagles during non-drought periods also (Klöcker et al. 2006; Read and Wilson 2004; Rees et al. 2019a). In keeping with this idea, previous studies conducted in arid regions where kangaroos have irrupted in the absence of dingoes have found that kangaroo is a frequent dietary item of wedge-tailed eagles during both drought and non-drought periods (Brooker and Ridpath 1980; Leopold and Wolfe 1970; Parker et al. 2007; Sharp et al. 2002b).

Consistent with previous studies on eagle diets in arid Australia, bearded dragons were frequent prey items for eagles (Parker et al. 2007; Sharp 1997; Sharp et al. 2002b). However, both the frequency of dragons at eagle nests and their abundance were greater on the side of the fence where dingoes were rare. This finding provides evidence that the abundance of bearded dragons and in turn their frequency in eagle diets may increase in response to the suppression of dingoes. Why these lizards may benefit from dingo exclusion is unclear.

One plausible hypothesis to explain the difference in bearded dragon abundance across the dingo fence is that they benefit from lower rates of predation because they are frequently preyed upon by dingoes (Cupples et al. 2011; Woinarski et al. 2017). However, that bearded dragons are also preyed upon by red foxes and feral cats which are also more abundant where dingoes are rare goes against this hypothesis (Cupples et al. 2011; Feit et al. 2019; Woinarski et al. 2017). Another hypothesis to explain the difference in bearded dragon numbers on either side of the dingo fence is that they have benefitted from the encroachment of woody shrubs which has occurred on the side of the fence where dingoes are rare (Gordon et al. 2017a). Bearded dragons make extensive use of above-ground perches such as live and dead shrubs for their thermoregulation (Melville and Schulte Ii 2001). Thus, it is conceivable that their populations may benefit from increased availability of perches if these perches also provide them with protection from predators.

Taxa encountered at low frequencies at eagle nests included dingoes, foxes and cats. The presence of mammalian predators at eagle nests is consistent with previous studies which have found that eagles prey on red foxes and feral cats (Brooker and Ridpath 1980; Debus and Rose 1999; Parker et al. 2007; Sharp et al. 2002a). These findings and, in particular, our discovery of the remains of a dingo pup at a nest highlight that eagles engage in “super-predation” whereby they kill and consume other higher-order predators (Lourenço et al. 2018). Thus, we hypothesize that eagles may exert some top-down control on the populations and behaviour of these mammalian predators, particularly if they prey on juveniles. Lourenço et al. (2018) suggested that such super-predation may have complex repercussions for the organization of ecosystems because other predators, whilst being a potential source of food, are also dangerous and competitors for food resources. Following this line of thinking, eagles that engage in super-predation behaviour must trade-off the risk of attacking dangerous prey with the benefits accrued by obtaining food and removing potential threats and competitors for food (Lourenço et al. 2018).

That the presence/absence of dingoes can influence the diet of wedge-tailed eagles highlights how pervasive the effects of trophic cascades stemming from the removal of apex predators’ on ecosystems can be. Apex predators’ effects on ecosystems are far reaching because they directly and indirectly influence the strength of trophic and competitive interactions between numerous species and also the strength of the interactions that vertebrates and plants have with abiotic processes. For example, in our Strzelecki Desert study region, removal of dingoes has been linked to measurable changes in ecosystem composition and function resulting from shifts in interaction strength between dingoes and kangaroos (Caughley et al. 1980), between kangaroos and their forage species (Morris and Letnic 2017), between kangaroos and soil nutrients (Morris and Letnic 2017) and between kangaroos and eagles (this study). A similar cascade of shifts in the balance of interspecific interactions has also stemmed from relaxation of dingoes’ suppressive effects on populations of red foxes and feral cats (Gordon et al. 2017a; Gordon and Letnic 2016; Rees et al. 2019b). The net result of these perturbations between dyads of interacting species at multiple trophic levels has been the reorganization of ecosystems on each side of the dingo fence into separate ecological universes with the same species pool but organized in a starkly different way.

## Data Availability

The datasets generated during and/or analysed during the current study are available from the corresponding author on reasonable request.

## References

[CR1] Beschta RL, Ripple W (2006). River channel dynamics following extirpation of wolves in northwestern Yellowstone National Park, USA. Earth Surface Processes Landforms: J British Geomorphological Res Group.

[CR2] Brooker MG, Ridpath M (1980). The diet of the wedge-tailed eagle, Aquila audax, in Western Australia. Wildl Res.

[CR3] Caughley G, Grigg G, Caughley J, Hill GJ (1980). Does dingo predation control the densities of kangaroos and emus?. Wildl Res.

[CR4] Clarke KR (1993). Non-parametric multivariate analyses of changes in community structure. Aust J Ecol.

[CR5] Clarke KR, Gorley RN (2001) PRIMER v.5: Users manual/Tutorial. PRIMER-E, Plymouth, UK

[CR6] Contos P, Letnic M (2019). Top-down effects of a large mammalian carnivore in arid Australia extend to epigeic arthropod assemblages. J Arid Environ.

[CR7] Corbett L, Hertog T, Estbergs J (2014). Diet of 25 sympatric raptors at Kapalga, Northern Territory, Australia 1979–89, with data on prey availability. Corella.

[CR8] Crowther MS, Fillios M, Colman N, Letnic M (2014). An updated description of the A ustralian dingo (C anis dingo M eyer, 1793). J Zool.

[CR9] Cupples JB, Crowther MS, Story G, Letnic M (2011). Dietary overlap and prey selectivity among sympatric carnivores: could dingoes suppress foxes through competition for prey?. J Mammal.

[CR10] Debus S (2017) Australasian Eagles and Eagle-like Birds. CSIRO PUBLISHING

[CR11] Debus S, Rose A (1999). Notes on the diet of the wedge-tailed eagle ‘Aquila audax’. Australian Bird Watcher.

[CR12] Estes JA (2011). Trophic Downgrading Planet Earth Sci.

[CR13] Feit B, Feit A, Letnic M (2019). Apex predators decouple population dynamics between mesopredators and their prey. Ecosystems.

[CR14] Fisher AG, Mills CH, Lyons M, Cornwell WK, Letnic M (2021). Remote sensing of trophic cascades: multi-temporal landsat imagery reveals vegetation change driven by the removal of an apex predator. Landscape Ecol.

[CR15] Fleming P, Corbett L, Harden B, Thomson P (2001). Managing the impacts of dingoes and other wild dogs.

[CR16] Fuentes E, Olsen J (2015). Observations of the killing of large macropods by wedge-tailed eagles ‘Aquila audax’. Australian Field Ornithol.

[CR17] Gordon CE, Letnic M (2016). Functional extinction of a desert rodent: implications for seed fate and vegetation dynamics. Ecography.

[CR18] Gordon CE, Eldridge DJ, Ripple WJ, Crowther MS, Moore BD, Letnic M (2017). Shrub encroachment is linked to extirpation of an apex predator. J Anim Ecol.

[CR19] Gordon CE, Moore BD, Letnic M (2017). Temporal and spatial trends in the abundances of an apex predator, introduced mesopredator and ground-nesting bird are consistent with the mesopredator release hypothesis. Biodivers Conserv.

[CR20] Harder M (2000). Diet and breeding biology of the wedge-tailed Eagle Aquila audax at three nests in northeastern New South Wales. Corella.

[CR21] Klöcker U, Croft DB, Ramp D (2006). Frequency and causes of kangaroo–vehicle collisions on an Australian outback highway. Wildl Res.

[CR22] Lee JS, Letnic M, Mills CH (2021). Diet and occurrences of the letter-winged kite in a predation refuge. Sci Nature.

[CR23] Leopold AS, Wolfe T (1970). Food habits of nesting wedge-tailed eagles, Aquila audax, in south-eastern Australia. CSIRO Wildlife Research.

[CR24] Letnic M, Crowther M (2020). Pesticide use is linked to increased body size in a large mammalian carnivore. Biol J Lin Soc.

[CR25] Letnic M, Koch F (2010). Are dingoes a trophic regulator in arid Australia? A comparison of mammal communities on either side of the dingo fence. Austral Ecol.

[CR26] Letnic M, Koch F, Gordon C, Crowther MS, Dickman CR (2009). Keystone effects of an alien top-predator stem extinctions of native mammals. Proceed Royal Soc B Biol Sci.

[CR27] Letnic M, Fillios M, Crowther MS (2012). Could direct killing by larger dingoes have caused the extinction of the thylacine from mainland Australia?. PLoS ONE.

[CR28] Lourenço R (2018). Why do top predators engage in superpredation? From an empirical scenario to a theoretical framework. Oikos.

[CR29] Lyons MB, Mills CH, Gordon CE, Letnic M (2018). Linking trophic cascades to changes in desert dune geomorphology using high-resolution drone data. J R Soc Interface.

[CR30] McKnight TL (1969) Barrier fencing for vermin control in Australia. Geographical Review:330–347. 10.2307/213480

[CR31] McKnight TL (1970). Biotic influences on Australian pastoral land use. Yearbook Assoc Pacific Coast Geographers.

[CR32] Melville J, Schulte Ii JA (2001). Correlates of active body temperatures and microhabitat occupation in nine species of central Australian agamid lizards. Austral Ecol.

[CR33] Morris T, Letnic M (2017). Removal of an apex predator initiates a trophic cascade that extends from herbivores to vegetation and the soil nutrient pool. Proceed Royal Soc B: Biol Sci.

[CR34] Newsome TM, Spencer EE (2021). Megafires attract avian scavenging but carcasses still persist. Divers Distrib.

[CR35] Newsome A, Catling P, Cooke BD, Smyth R (2001). Two ecological universes separated by the dingo barrier fence in semi-arid Australia: interactions between landscapes, herbivory and carnivory, with and without dingoes. Rangeland J.

[CR36] Olsen J, Judge D, Fuentes E, Rose A, Debus SJ (2010). Diets of wedge-tailed eagles (Aquila audax) and little eagles (Hieraaetus morphnoides) breeding near Canberra, Australia. J Raptor Res.

[CR37] Olsen J, Cooke B, Trost S, Judge D (2014). Is wedge-tailed eagle, Aquila audax, survival and breeding success closely linked to the abundance of European rabbits, Oryctolagus cuniculus?. Wildl Res.

[CR38] Parker BD, Hume ID, Boles WE (2007) Diet of breeding wedge-tailed eagles Aquila audax in south-central Queensland.

[CR39] Prugh LR (2009). The rise of the mesopredator. Bioscience.

[CR40] Read J, Wilson D (2004). Scavengers and detritivores of kangaroo harvest offcuts in arid Australia. Wildl Res.

[CR41] Rees JD, Kingsford RT, Letnic M (2019). Changes in desert avifauna associated with the functional extinction of a terrestrial top predator. Ecography.

[CR42] Rees JD, Rees GL, Kingsford RT, Letnic M (2019). Indirect commensalism between an introduced apex predator and a native avian predator. Biodivers Conserv.

[CR43] Rees JD, Crowther MS, Kingsford RT, Letnic M (2020). Direct and indirect effects of carrion subsidies in an arid rangeland: carrion has positive effects on facultative scavengers and negative effects on a small songbird. J Arid Environ.

[CR44] Ridpath M, Brooker MG (1986). The breeding of the wedge-tailed eagle Aquila audax in relation to its food supply in arid Western Australia. Ibis.

[CR45] Ridpath M, Brooker MG (1987). Sites and spacing of nests as determinants of wedge-tailed eagle breeding in arid Western Australia. Emu.

[CR46] Ripple WJ, Beschta RL (2004). Wolves and the ecology of fear: can predation risk structure ecosystems?. Bioscience.

[CR47] WJ Ripple et al (2014) Status and ecological effects of the world’s largest carnivores. Science 34310.1126/science.1241484 10.1126/science.1241484 24408439

[CR48] Sharp A (1997). Notes on the breeding season diet of the wedge-tailed eagle ‘Aquila audax’ in Idalia National Park, South-central Queensland.. Sunbird: J Queensland Ornithol Soc.

[CR49] Sharp A, Norton M, Marks A (2001). Breeding activity, nest site selection and nest spacing of wedge-tailed eagles, Aquila audax, in western New South Wales. Emu.

[CR50] Sharp A, Gibson L, Norton M, Marks A, Ryan B, Semeraro L (2002). An evaluation of the use of regurgitated pellets and skeletal material to quantify the diet of wedge-tailed Eagles, Aquila audax. Emu-Austral Ornithology.

[CR51] Sharp A, Gibson L, Norton M, Ryan B, Marks A, Semeraro L (2002). The breeding season diet of wedge-tailed eagles (Aquila audax) in western New South Wales and the influence of rabbit calicivirus disease. Wildl Res.

[CR52] Silva LM, Croft DB (2007). Nest-site selection, diet and parental care of the wedge-tailed eagle Aquila audax in western New South Wales. Corella.

[CR53] Winkel P (2007). Feeding ecology of the wedge-tailed eagle Aquila audax in north-west Queensland: interactions with lambs. Corella.

[CR54] Woinarski JC, South SL, Drummond P, Johnston GR, Nankivell A (2017). The diet of the feral cat (Felis catus), red fox (Vulpes vulpes) and dog (Canis familiaris) over a three-year period at Witchelina Reserve, in arid South Australia. Australian Mammal.

[CR55] AF Zuur EN Ieno N Walker AA Saveliev GM Smith (2009) Mixed effects models and extensions in ecology with R Springer. Sci Business Media10.1007/978-0-387-87458-6_5

